# Fatal lymphatic-dominant progression in metastatic prostate cancer initially presenting with a clinically suspected inguinal node metastasis: a case report

**DOI:** 10.3389/fonc.2026.1751798

**Published:** 2026-04-01

**Authors:** Jian Yang, Yonglin Huang, Bin Zeng

**Affiliations:** 1 Department of Urology, Zigong First People’s Hospital, Zigong, Sichuan, China; 2 Department of Urology, Rongxian People’s Hospital, Zigong, Sichuan, China; 3 Department of Urology, Institute of Urology and National Clinical Research Center for Geriatrics, West China Hospital of Sichuan University, Chengdu, Sichuan, China

**Keywords:** case report, drug resistance, inguinal node metastasis, lymphatic-dominant progression, prostate cancer

## Abstract

Prostate cancer presenting with a clinically suspected inguinal lymph node metastasis (ILNM) is an exceptionally rare and aggressive disease variant. We report a case of a patient with metastatic hormone-sensitive prostate cancer (mHSPC) who presented with an isolated inguinal mass and subsequently developed fatal lymphatic-dominant progression despite standard intensive systemic therapy and an initial deep PSA response. A 68-year-old male patient presented with a painless right groin mass. Imaging and biopsy confirmed high-volume mHSPC (PSA 776.0 ng/mL, Gleason score 8) with suspected inguinal, pelvic, and bone metastases. Treatment with androgen deprivation therapy (ADT) and abiraterone acetate was initiated, achieving a PSA nadir of <0.090 ng/mL. Approximately nine months later, he developed refractory lower limb and scrotal edema, bilateral hydronephrosis, and renal impairment. Despite comprehensive supportive care including ureteral stenting, his condition deteriorated rapidly, resulting in death. This case highlights a rare clinical scenario of lymphatic-dominant progression occurring despite excellent systemic biochemical control. It underscores the need for vigilant monitoring of regional lymphatic complications and early multidisciplinary intervention in high-risk mHSPC patients presenting with non-typical metastases.

## 1 Introduction

Prostate cancer (PCa) is one of the most common malignant tumors in the male urinary and reproductive system, with a significant social impact. PCa is the second (after lung cancer) male malignant tumor in the world, according to the latest reports (1). For PCa, its clinical symptoms most commonly manifest as obstructive symptoms, such as slow urine flow, urgency, frequency, thin urine stream, incomplete emptying of the bladder, and difficulty in urination (2). Prostate cancer typically metastasizes to regional lymph nodes and bones via well-defined lymphatic pathways (3). Inguinal lymph node metastasis (ILNM) as an initial presentation is exceedingly rare, with an incidence of less than 1%, and is often associated with high-grade disease and poor prognosis (4). The mechanisms may involve aberrant lymphatic drainage or direct spread along the spermatic cord (5).

The standard first-line therapy for high-volume metastatic hormone-sensitive prostate cancer (mHSPC) is androgen deprivation therapy (ADT) combined with novel hormonal agents like abiraterone acetate. While this regimen significantly improves outcomes, heterogeneous responses and patterns of progression are observed. We present a case of mHSPC with initial clinically suspected ILNM that achieved a deep PSA response but subsequently progressed with severe regional lymphatic complications, underscoring the diagnostic and therapeutic challenges in managing such atypical presentations.

## 2 Case report

### 2.1 Patient information

A 68-year-old man presented on July 11, 2024, with a several-day history of a painless, palpable mass in the right groin. The patient reported being previously healthy, denying any history of chronic medical conditions such as hypertension or diabetes mellitus. He had no regular medication use. He was a non-smoker and denied significant alcohol use. His family history was non-contributory for prostate or other malignancies. He was 173 cm in height and weighed 58 kg, resulting in a body mass index (BMI) of 19.4 kg/m². At presentation, his Eastern Cooperative Oncology Group (ECOG) performance status was 1.

### 2.2 Clinical presentation, diagnostic findings, and initial staging

**Figure 1:**
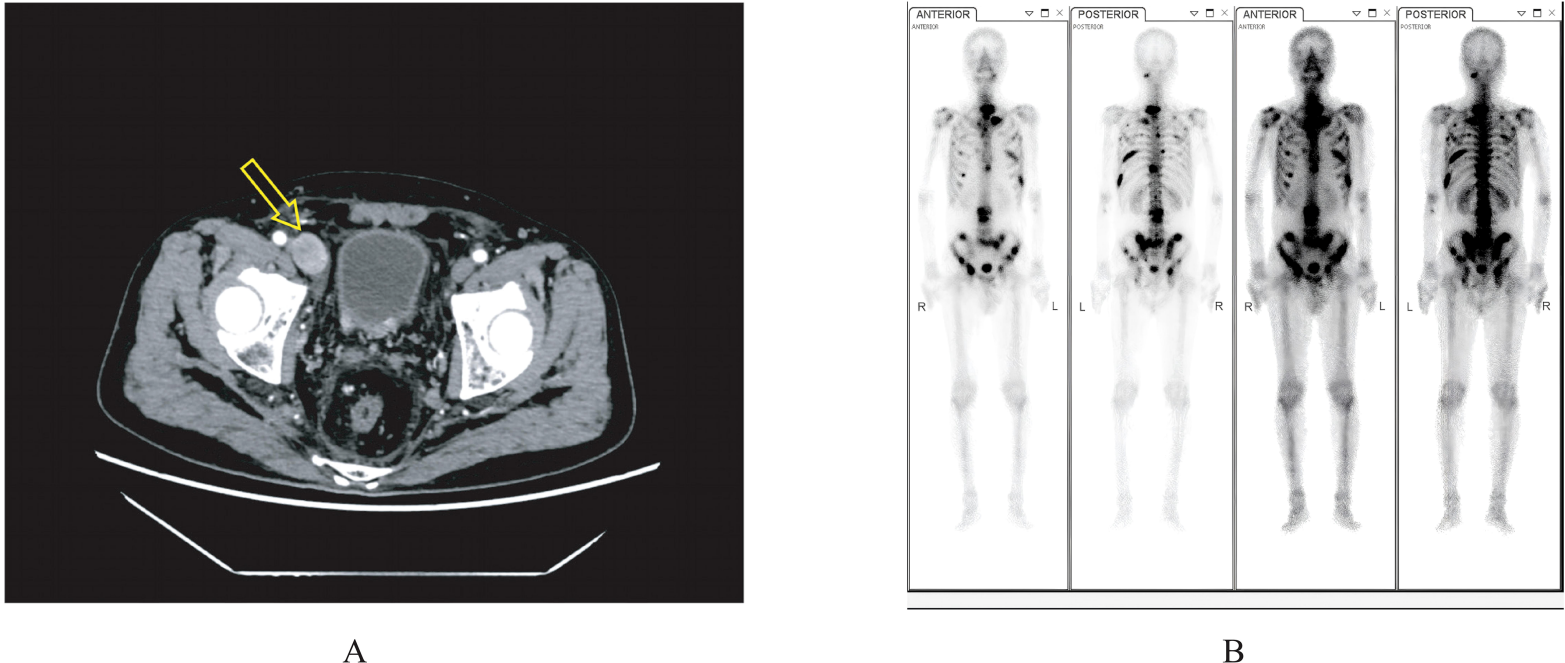
**(A)** Enhanced computed tomography (CT) scan of the abdomen and pelvis, axial view, performed on July 15, 2024, demonstrating right inguinal lymphadenopathy (yellow arrow). **(B)** Anterior and posterior whole-body skeletal scintigraphy with 99mTc-methylene diphosphonate (MDP), performed on July 22, 2024, showing multiple foci of increased radiotracer uptake consistent with bone metastases.

**Figure 2:**
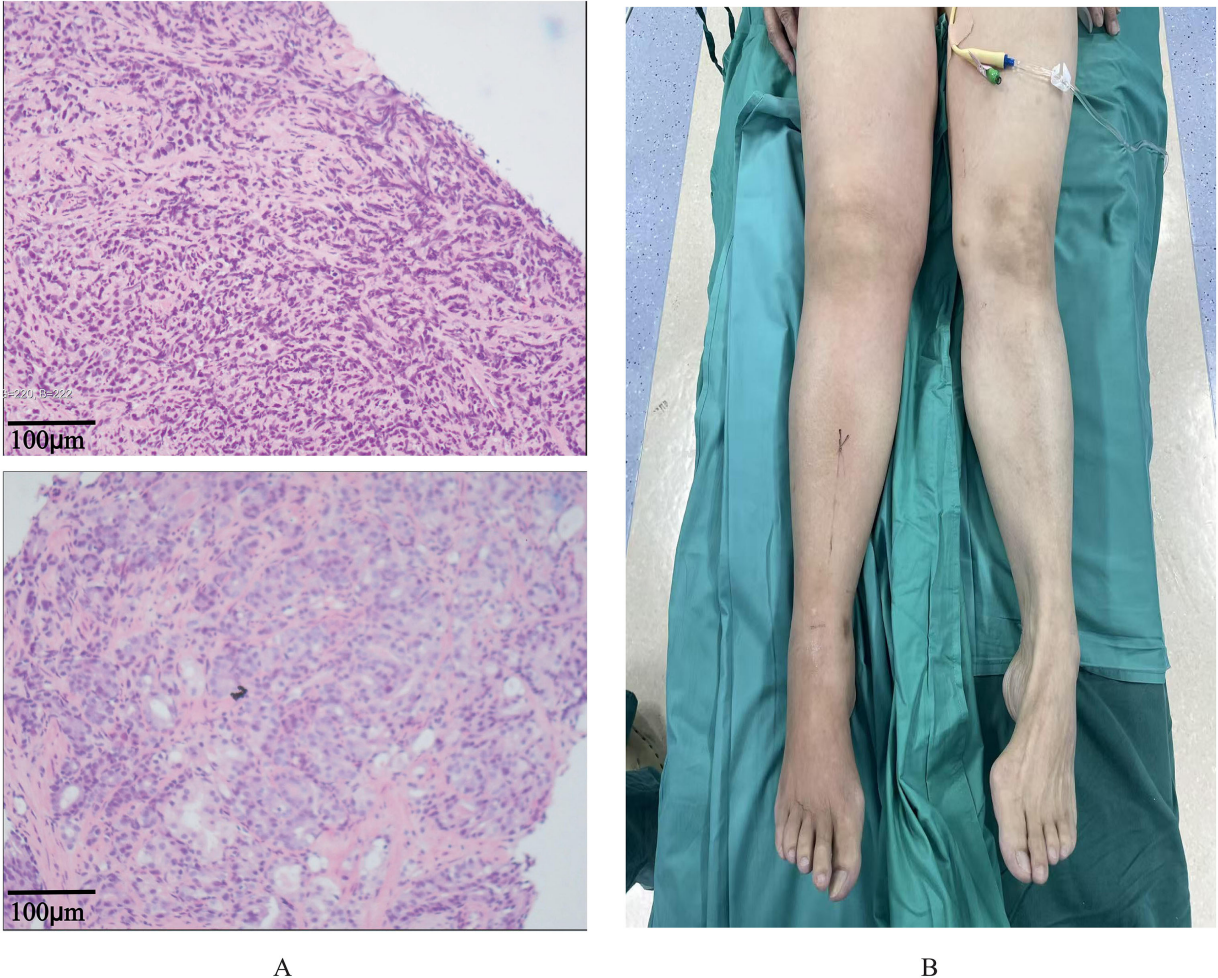
**(A)** Hematoxylin-eosin stain of prostate biopsy specimen. **(B)** Patient picture of right lower limb edema.

He reported no lower urinary tract symptoms or bone pain. Physical examination revealed a hard, fixed, non-mobile mass measuring approximately 3.0 cm × 3.5 cm in the right inguinal region. The distal pulses of the right lower limb were normal, and no scrotal or lower limb edema was initially detected. Admission laboratory tests (July 15, 2024) showed a total prostate-specific antigen (PSA) level of 776.0 ng/mL (free-to-total PSA ratio: 0.14). Other relevant markers included: CEA 33.05 ng/mL, CA125 17.8 U/mL, AFP 4.92 ng/mL, and CA19-9 <0.30 U/mL. Serum electrolytes, complete blood count, coagulation profile, liver and renal function tests were within normal limits. Chest X-ray and electrocardiogram were unremarkable. Serum testosterone level was within the normal adult male range prior to treatment initiation. Contrast-enhanced CT of the abdomen and pelvis (July 15, 2024) revealed irregular enlargement of the prostate with adjacent bladder wall thickening, multiple enlarged lymph nodes in the right inguinal, pelvic, and retroperitoneal regions, and osteoblastic lesions in the left ninth rib, L3, and S1 vertebral bodies, suggestive of metastatic disease (**Figure 1A**). Subsequently, a whole-body bone scintigraphy (July 22, 2024) confirmed extensive skeletal metastases involving the cervical spine, multiple thoracic/lumbar vertebrae, sternum, bilateral ribs, left clavicle, bilateral scapulae, pelvic bones, and the proximal right humerus (**Figure 1B**). Transrectal ultrasound-guided prostate biopsy confirmed acinar adenocarcinoma with a Gleason score of 8 (4 + 4), ISUP Grade Group 4 (**Figure 2A**). Based on the presence of clinically suspected lymph node metastasis and extensive bone disease (>4 lesions), the disease was definitively classified as high-volume mHSPC according to the CHAARTED criteria. Notably, a biopsy of the inguinal mass was considered but ultimately not performed because of its deep anatomical location and its proximity to vascular structures. There was a relatively high risk of bleeding during puncture and damage to surrounding important tissues and organs. After communicating with the patient and their family, the patient refused to undergo a puncture biopsy. Its metastatic nature was inferred based on imaging features and subsequent treatment responses.

### 2.3 Therapeutic intervention and initial response

**Figure 3:**
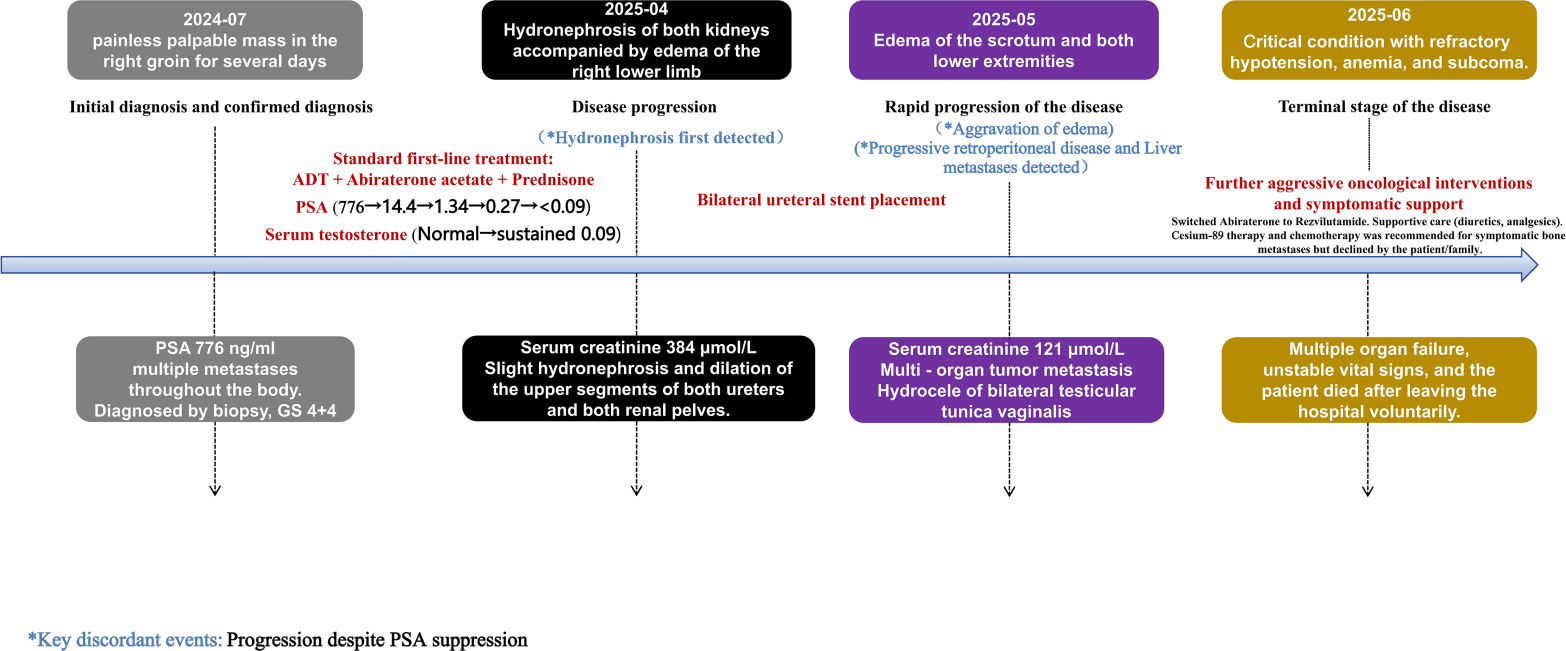
The timeline of key events for this case.

Following diagnosis, the patient commenced standard first-line therapy in late July 2024 with subcutaneous goserelin (an LHRH agonist, 3.6 mg monthly) combined with oral abiraterone acetate (1000 mg daily) and prednisone (5 mg daily). Zoledronic acid was administered to prevent skeletal-related events. Treatment was well-tolerated initially, with routine monitoring showing no significant hypertension, hypokalemia, or hepatotoxicity. The inguinal mass decreased in size on physical exam. Serial PSA and testosterone measurements demonstrated an excellent and sustained biochemical response: PSA declined from 776.0 ng/mL to 14.4 ng/mL (August 2024), 1.34 ng/mL (September), 0.27 ng/mL (October), and reached a nadir of <0.090 ng/mL by November 2024, which was maintained through March 2025. Serum testosterone was suppressed to castration levels (0.09 nmol/L) from August 2024 onward (**Figure 3**).

### 2.4 Diagnostic assessment and reasoning upon progression

In April 2025, approximately nine months after treatment initiation, the patient developed bilateral lumbar pain. Ultrasound (April 19) and CT (April 20) revealed new-onset bilateral hydronephrosis and ureteral dilation, with serum creatinine rising to 111 μmol/L. The CT also noted a marked increase in the number of bone lesions but confirmed reduction in the size of inguinal/retroperitoneal lymphadenopathy. By late April 2025, renal function acutely worsened (creatinine 384 μmol/L). A comprehensive diagnostic workup was pursued to elucidate the cause of the hydronephrosis and the subsequent development of progressive edema, which began in the right lower limb (**Figure 2B**) and progressed to involve the scrotum and both lower limbs by May 2025. This workup systematically ruled out common alternative etiologies: bilateral lower extremity venous Doppler ultrasound (May 22) showed significant subcutaneous edema but no deep vein thrombosis; serum albumin levels were repeatedly within normal range (e.g., 43.1 g/L on May 19), arguing against generalized edema due to hypoproteinemia; and clinical assessment did not suggest heart failure as a primary cause. Concurrently, to investigate the hydronephrosis and renal failure, serial CT imaging (April 25, May 12, May 20) demonstrated progressive soft tissue infiltration and obscuration of the pelvic and retroperitoneal fat planes, with encasement of the ureters and development of perinephric stranding. A cystoscopy with attempted bilateral ureteroscopy on April 27, 2025, provided direct visual evidence, revealing extensive necrotic tissue and stenosis at both ureteral orifices that prevented stent passage. Based on the temporal association with known metastatic prostate cancer, the corroborative imaging findings of retroperitoneal progression despite systemic PSA control, the direct endoscopic findings of malignant-appearing obstruction, and the exclusion of other common causes, the leading diagnosis was malignant lymphatic infiltration and ureteral obstruction by tumor.

### 2.5 Follow-up, outcomes, and end-of-life course

After multidisciplinary discussion, successful bilateral ureteral stent placement was achieved, leading to a temporary improvement in renal function (creatinine decreased from 384 to 121 μmol/L). However, renal function gradually declined again over the ensuing weeks. Despite maximal supportive care, including diuretics and attempts to switch hormonal therapy (specifically, consideration of revilutamide (a novel oral androgen receptor inhibitor with a low incidence of fluid retention (6)) was given due to suspicion of abiraterone-related fluid retention (7)), the patient’s condition deteriorated. The edema worsened, and he developed anuria in June 2025, with creatinine peaking at 1270.4 μmol/L, necessitating the initiation of dialysis. Of note, at the time liver metastases were first detected on CT (May 20, 2025), PSA remained suppressed at <0.090 ng/mL (confirmed on May 19, 2025), underscoring the profound discordance between biochemical control and disease progression. Further aggressive oncological interventions, specifically chemotherapy (docetaxel) or radionuclide therapy (e.g., Strontium-89), were deemed not feasible or were declined by the family due to his critically impaired renal function, poor overall performance status (ECOG 3-4), and patient/family preferences after detailed consultation. Subsequently, the patient developed hypotension (the systolic blood pressure fluctuated between 60–65 mmHg), anemia, and a diminished level of consciousness. His family opted for discharge with hospice care and died shortly thereafter, approximately 11 months from diagnosis. The most likely terminal event was progressive tumor-related multi-organ failure, culminating in refractory renal failure. The timeline of key events, incorporating PSA and renal function trends, is summarized in **Table 1** and **Figure 3**.

**Table 1:** Clinical timeline and interventions for this metastatic prostate. Cancer Case.

Date	Event	PSA (ng/mL)	Testosterone (nmol/L)	Imaging/pathology findings	Creatinine (µmol/L)	Interventions	Key notes
2024-07-11	Hospital admission for a painless right groin mass.	776	Not recorded	CT: Irregularly enlarged prostate with adjacent bladder wall thickening/enhancement; multiple enlarged lymph nodes in right groin/pelvis/retroperitoneum; bone destruction (L3, S1, etc.). CXR, ECG: Normal. Biopsy: Prostatic acinar adenocarcinoma, GS 4 + 4 (ISUP Grade 4). Bone Scan: Widespread skeletal metastases.	Normal (value not specified)	Initial Therapy: Goserelin (ADT) + Abiraterone Acetate + Prednisone initiated. Zoledronic acid for bone protection	Diagnosis established and Extensive metastases confirmed
2024-08-20 → 2025-03-20	Regular monthly follow-ups, disease well-controlled.	14.4 → <0.09	0.09 (sustained)	Not recorded	Normal (value not specified)	Continued ADT + Abiraterone + Prednisone.	Treatment initiated Good response
2025-04-19/20	Imaging shows hydronephrosis and progressive bone disease.	<0.09	0.09	Ultrasound/CT: Bilateral hydronephrosis & proximal ureteral dilation; left distal ureteral stone. Skeletal metastases increased in number/size compared to prior.	111	Continued first-line therapy.	*Hydronephrosis first detected
2025-04-25/26	Presents with bilateral flank pain and acute kidney injury.	Not recorded	Not recorded	CT: Worsening bilateral hydronephrosis, perinephric stranding. New bladder mass noted. Multiple masses in bladder trigone; bilateral ureteral orifices obscured by necrotic tissue and severely stenotic.	384	Cystoscopy + Bilateral Ureteral Stent Placement.	Cystoscopy/stent attempt
2025-05-12/19	Admission for severe scrotal and bilateral lower limb edema evolved from right lower limb edema	<0.09	0.09	CT: New/worsening multiple liver metastases; blurred pelvic fat planes/tortuous vessels; scrotal & bilateral hydroceles; worsening subcutaneous edema.	121	Therapy Change: Abiraterone discontinued. Switched to Rezvilutamide. Supportive care (diuretics, analgesics). Cesium-89 therapy was recommended for symptomatic bone metastases but declined by the patient/family.	*Progressive retroperitoneal disease *Liver metastases detected (The PSA level remains suppressed, but new metastatic lesions are found)
2025-06-08	Anuria, end-stage renal failure. Multi-organ failure.	Not recorded	Not recorded	Not Apply	1270.4	Initiated Hemodialysis. Chemotherapy (e.g., docetaxel) was considered but not tolerated due to the patient’s poor overall condition and rapid clinical decline.	Renal failure
Post 2025-06-10	Critical condition with refractory hypotension, anemia, and subcoma.	Not recorded	Not recorded	Not Apply	Not recorded	Life-sustaining measures (vasopressors, oxygen) ineffective. Patient/family declined ICU transfer and further aggressive interventions.	Tumor-related multi-organ failure
Post Discharge	Patient deceased.	–	–	–	–	–	Death

*Key discordant events: Progression despite PSA suppression.

## 3 Discussion

This report describes a patient with high-volume mHSPC who presented with a clinically suspected ILNM—a rare occurrence reported in <1% of cases and associated with high-grade disease and poor prognosis (8). Previous case reports describe patients across a wide age range, often with very high PSA levels, and sometimes without typical urinary symptoms, which can lead to diagnostic delay (9). Our patient’s presentation aligns with these features (PSA >700 ng/mL, Gleason 8, no LUTS). Building on this foundation, a more detailed comparison with previously reported ILNM cases reveals both similarities and important distinctions. The published literature confirms that such patients consistently present with very high PSA levels (ranging from 35 to >500 ng/mL) and high-grade tumor (5, 10, 11). However, most historical reports predate the era of intensified androgen receptor pathway inhibitors and did not document the phenomenon of PSA-discordant lymphatic progression observed here. Regarding PSA-discordant progression, our case aligns with a growing recognition that mHSPC can exhibit heterogeneous responses, with some studies describing various radiographic progressions despite biochemical control (12–14). Literature on regional lymphatic complications in advanced prostate cancer remains sparse, but our case adds to this body of knowledge by documenting a clear ‘lymphatic-dominant’ syndrome—progressive lymphedema and ureteral obstruction in the absence of PSA rise.

The most striking aspect of this case is the dissociation between the excellent systemic biochemical response and the clinically aggressive regional progression. The patient achieved a deep and sustained PSA response (<0.09 ng/mL) with castrate-level testosterone and radiological regression of measurable lymphadenopathy, meeting standard criteria for initial treatment success. This biochemical and partial radiological response effectively rules out “primary resistance” to the administered hormonal therapy. Instead, the progression manifested almost exclusively as a “lymphatic-dominant” clinical syndrome—characterized by refractory lymphedema and ureteral obstruction due to retroperitoneal infiltration—without a concurrent significant rise in PSA. This pattern suggests a focal failure within a context of systemic control, possibly due to tumor heterogeneity or clonal evolution.

The observed “lymphatic-dominant” progression pattern invites speculation about potential underlying biological mechanisms, though our single case lacks the molecular data to prove causation. We present the following as hypotheses to guide future investigation. We hypothesize that pre-existing or emergent subclones with distinct properties may have driven this course. Potential mechanisms, worthy of investigation in future similar cases, could potentially involve enhanced Epithelial-to-Mesenchymal Transition (EMT), associated with increased migratory and invasive potential (15); might be explained by altered chemokine receptor expression, such as upregulation of CCR7 known to guide cells toward lymphatic vessels (16); or could reflect the acquisition of aggressive genomic alterations like the loss of tumor suppressors RB1 or TP53, which are linked to treatment resistance and more aggressive phenotypic variants (17, 18). A critical limitation of our report is the absence of molecular profiling of the progressing retroperitoneal disease, which would have been invaluable for testing these hypotheses.

This case offers crucial practical lessons for clinicians managing similar high-risk presentations. First, it underscores that in mHSPC, especially with non-typical initial features such as ILNM, progression can occur through mechanisms not reflected by PSA. New or worsening lymphedema or hydronephrosis should be recognized as potential “red flags” for regional advancement, necessitating immediate investigation. Second, a prompt and comprehensive diagnostic workup is imperative to rule out alternative causes (e.g., DVT, benign obstruction) and confirm malignant etiology, as demonstrated here. Third, management of such complications requires early and sustained multidisciplinary collaboration. Ureteral stenting is a vital palliative tool, but its benefit may be transient if tumor infiltration progresses, highlighting the need for concurrent effective systemic disease control, which was profoundly challenging in this case. Finally, for patients presenting with ultra-high-risk features (e.g., ILNM, extreme PSA, high-volume disease), the feasibility of upfront triple intensive therapy (ADT + novel hormonal agent + docetaxel chemotherapy) may warrant discussion in selected high-risk presentations (19, 20), though this patient’s rapid functional decline precluded such options. While randomized trials such as ARANOTE (19) (OS: HR = 0.68, 95% CI:0.57-0.80; P<0.001) and PEACE-1 (20) (OS: HR = 0.75, 95% CI:0.59-0.95; P = 0.017) have established the efficacy of triple intensive therapy (ADT + novel hormonal agent + docetaxel chemotherapy) has shown benefit in high-volume mHSPC, the application of such intensified regimens to patients with ultra-high-risk features like ILNM remains an area for clinical judgment and further study. Biopsy of accessible metastatic sites for molecular characterization should be strongly considered when feasible to guide therapy.

Based on the above analysis, we suggest that a more aggressive clinical management strategy be considered for this type of “ultra-high-risk” mHSPC patients. For newly diagnosed patients with features such as ILNM and extremely high PSA (>500 ng/mL), they should be considered as a subgroup requiring special attention. Meanwhile, biopsy of metastatic lesions (such as the inguinal lymph nodes in this case) should be sought at the time of diagnosis for comprehensive molecular typing to find biomarkers for guiding treatment. In addition, a highly vigilant monitoring mechanism must be established, and the assessment of the lymphatic system should be included in routine follow-up. Once early signs of lymphatic reflux obstruction appear, multidisciplinary team intervention should be initiated immediately.

## 4 Limitation

This report has several important limitations that should be considered when interpreting the findings. First, as a single retrospectively reported case, the findings cannot be generalized to all patients with mHSPC. Second, histopathological confirmation of the inguinal lymph node metastasis was not obtained. Biopsy was considered but deemed high-risk due to the node’s anatomical location adjacent to vascular structures, and the diagnosis of metastasis was inferred from imaging characteristics and treatment response. Third, and most importantly, no tissue sampling or molecular profiling was performed at the time of disease progression. Consequently, we lack the genomic or transcriptomic data that would be necessary to validate the biological hypotheses (e.g., RB1/TP53 loss, EMT activation) proposed in the discussion. Fourth, the patient’s rapid clinical deterioration limited our ability to pursue additional therapeutic options and to collect more detailed longitudinal data. These limitations caution against overgeneralization but highlight important directions for future research, particularly the need for prospective studies with serial biopsies to elucidate mechanisms of treatment resistance in patients with atypical metastatic presentations.

## 5 Conclusion

This case illustrates a rare but devastating lymphatic-dominant progression pattern in mHSPC, which can occur despite an excellent initial systemic biochemical response to intensified hormonal therapy. It underscores that ILNM at presentation may signal a high-risk disease course requiring heightened clinical vigilance for regional complications. Management should emphasize early multidisciplinary intervention, thorough diagnostic evaluation of new symptoms, and, where possible, pursuit of biomarker characterization to inform therapy in this challenging patient subgroup.

## Data Availability

The original contributions presented in the study are included in the article/supplementary material. Further inquiries can be directed to the corresponding author.
